# Rifampicin resistance mutations in the 81 bp RRDR of *rpoB* gene in *Mycobacterium tuberculosis* clinical isolates using Xpert^®^MTB/RIF in Kampala, Uganda: a retrospective study

**DOI:** 10.1186/1471-2334-14-481

**Published:** 2014-09-04

**Authors:** Gerald Mboowa, Carolyn Namaganda, Willy Ssengooba

**Affiliations:** Department of Medical Microbiology, School of Biomedical Sciences, College of Health Sciences, Makerere University, P.O Box 7072, Kampala, Uganda

**Keywords:** Rifampicin-resistance-determining region, RNA polymerase B gene, Multi-drug Resistant tuberculosis, Xpert^®^ MTB/RIF

## Abstract

**Background:**

Introduction of Xpert^®^ MTB/RIF assay has revolutionalised the diagnosis of tuberculosis (TB) by simultaneously detecting the bacteria and resistance to rifampicin (rif), a surrogate marker for multi-drug resistant TB (MDR-TB) as well as one of the principal first-line anti-tuberculosis drugs. In general, *rpoB* mutations can be found in 96.1% of rif-resistant *Mycobacterium tuberculosis* (MTB) strains worldwide and these mutations usually are located in a region at the 507-533^rd^ amino acid residuals (81 bp) in the MTB *rpoB* gene, which is referred to as Rifampicin-resistance-determining region (RRDR). In this study, we determined the frequency of MDR-TB in Kampala using Xpert^®^ MTB/RIF in comparison with the agar proportion method using Middlebrook 7H11and further determined the frequency of probes for different *rpoB* gene mutations using Xpert^®^ MTB/RIF assay in the 81 bp RRDR.

**Methods:**

A total of 1501 specimens received at Mycobacteriology laboratory, Makerere University for Xpert testing between May 2011 and May 2014 were analysed by Xpert^®^ MTB/RIF assay. Specimens that were positive for both MTB and rifampicin resistance were further subjected to a complete first line anti-mycobacterial drug susceptibility testing using Middlebrook 7H11 agar proportion method (APM).

**Results:**

Xpert^®^ MTB/RIF assay detected 313 MTB positive specimens and out of which 12 specimens had both MTB and rifampicin- resistance conferred by four different *rpoB* gene mutations in the 81 bp-RRDR of MTB, further one (1/12), specimen was found to be rifampicin mono-resistant on APM while the 11 were found to be MDR-TB. Probes associated with the observed rif- resistance were as follows: E (7/12), B (3/12), A (1/12), D (1/12) and no rif-resistance was associated with probe C. No specimen yielded rif-resistance associated with more than one probe failure (mutation combinations). Probe D was associated with rifampicin mono-resistant.

**Conclusions:**

MDR-TB was at 3.5% in the studied population. Mutations associated with Probe E (58%) also known as codons 531and 533 are the commonest *rpoB* gene mutation identified by Xpert^®^ MTB/RIF assay in this setting and mutations identified by probe E of the assay, turned out to be MDR-TB strains by agar proportion method antimicrobial susceptibility testing. No mutation was detected in the codon 522.

**Electronic supplementary material:**

The online version of this article (doi:10.1186/1471-2334-14-481) contains supplementary material, which is available to authorized users.

## Background

Introduction of Xpert^®^ MTB/RIF assay (Cepheid, USA) has revolutionalised the diagnosis of tuberculosis (TB) by simultaneously detecting the bacteria and resistance to rifampicin (rif) [1, 2], one of the principal first-line anti-tuberculosis drugs, which inhibits DNA-directed RNA synthesis of *Mycobacterium tuberculosis* (MTB) proteins by binding to the β-subunit of bacterial DNA dependent RNA polymerase (rpoB) enzyme. In general, *rpoB* mutations can be found in 96.1% of rif-resistant MTB strains worldwide and these mutations are usually located in a region at the 507- 533^th^ amino acid residuals (81 bp) in the *rpoB* gene, which is often called rifampicin-resistance determining region (RRDR) [3]. Rifampicin resistance is a valuable surrogate marker of multi drug resistant (MDR) TB [4].

Uganda ranked 16^th^ among 22 high TB burden nations and WHO, 2012 report showed that the prevalence of MDR-TB was 1.4% and 12% among new and previously treated TB cases, respectively. In Uganda, a total of 1406 cases tested for MDR-TB with 89 laboratory confirmed cases and only 41 cases were started on treatment [5]. The biological features of bacteria, including drug resistance; usually differ in diverse geographical regions [6–10]. Data on prevalence of *rpoB* gene mutations in Uganda is limited, therefore this study was set to provide baseline data on these mutations using Xpert^®^ MTB/RIF assay. Clinical observations suggest that not only detection of the presence but also identification of the nature of *rpoB* mutation is needed for accurate diagnosis of resistance to rifampicin [11]. Therefore, in this study; we investigated the rif-resistant frequency of MTB strains isolated from specimens of patients suspected to have TB in Kampala-Uganda and the dominant site mutation types at RRDR in the *rpoB* gene of rif-resistant MTB isolates using Xpert^®^ MTB/RIF assay.

## Methods

### Ethical issues

The study projects referring the specimens were approved by the Research and Ethics Committee of the School of Biomedical Sciences of Makerere University, as well as the Uganda National Council for Science and Technology (UNCST). Parents/guardians were informed about the study and written informed consent was obtained from the parents or legal guardians, were applicable.

### Clinical specimens

**Figure 1:**
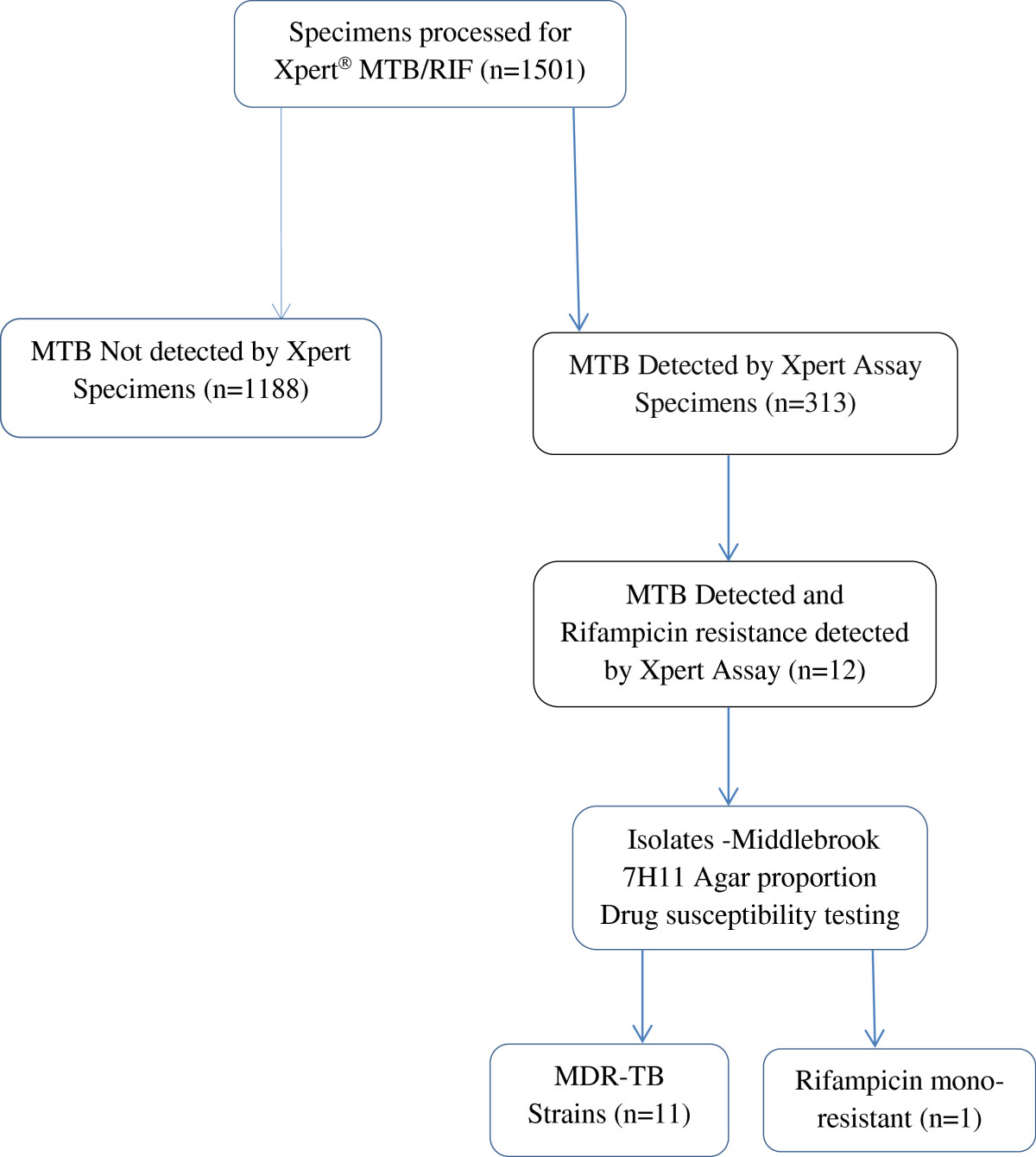
**Flow**-**chart of specimen analysis**.

In total, 1501 specimens were included in the study. They originated from patients with suspected tuberculosis infections and were sent for routine mycobacteriology diagnostics between May 2011 and May 2014 at Mycobacteriology (BSL-3) laboratory, Makerere University. This is the only TB culture laboratory located in Mulago Hospital complex, a national referral as well as a university teaching hospital serving both urban and peri-urban population of about two million people. The studies referring these specimens had protocols approved by the School of Biomedical Sciences ethics committee at College of Health Sciences, Makerere University.When these specimens were received at the laboratory, all patient critical details were capture and specimen characteristics (volume and consistence) were recorded in laboratory data collection forms and later captured in access restricted computer-based database. These specimens included sputa, cerebral spinal fluid (CSF), pleural fluid, gastric aspirate and nasopharyngeal aspirate. Tuberculosis diagnostic requests ranged from microscopy, culture and Xpert^®^ MTB/RIF assay. In addition to requested routine analyses, we did perform first-line anti-TB drug susceptibility testing on all specimens that were positive for both MTB and rif –resistance by Xpert^®^ MTB/RIF assay, as shown in Figure 1.

Specimens were decontaminated using N-acetyl-L-cysteine (NALC) - sodium hydroxide (NaOH) except cerebral spinal fluid (CSF). After concentration by centrifugation at 3000 g for 15 minutes, the sediment was resuspended in 1.5 ml of 0.5 M phosphate buffer (pH 6.8) and inoculated in MGIT-7H9 broth supplemented with oleic acid-albumin-dextrose-catalase (OADC) and PANTA (Becton Dickinson). This was incubated using MGIT™ Bactec 960 instrument (Becton-Dickinson and Company, Sparks MD) as well as Loewenstein-Jensen (LJ) medium at 37°C. Concentrated Smears were prepared from this suspension, stained with Auramine-O-phenol stain and visualized with a fluorescence microscope (magnification X200 and X400). The remaining sediment was stored frozen for other preferred tests. LJ and MGIT cultures were incubated for up to 8 and 6 weeks respectively. Ziehl- Neelsen (ZN) staining for acid fast bacilli was performed on suspicious growth on LJ and MGIT™ instrument positive tubes. Capilia was done to confirm MTB complex.

### Agar proportion method for phenotypic drug susceptibility testing

The agar proportion method using Middlebrook 7H11 was performed as per established standard procedures and calculations for resistance also made as recommended in standard. *Mycobacterium tuberculosis* H37Rv (sensitive to all the antituberculosis agents) was used as reference control strain. The drugs were used at critical concentrations.

### Xpert^®^MTB/RIF assay

Both processed sediments and unprocessed specimens were used for the Xpert^®^ MTB/RIF assay. This was done according to the manufacturer’s instructions.

### Statistical methods

Test results were based on comparison of rifampicin resistance derived from the Xpert^®^ MTB/RIF assay using Xpert^®^ MTB/RIF assay G4 version 5 and Xpert^®^ MTB/RIF Assay G3 version 3. All the Rif-resistant-TB cases were detected by Xpert^®^ MTB/RIF G4 version 5. Results for Xpert^®^ MTB/RIF determined rifampicin resistance were compared with APM results as the reference comparator to for agreement and concordance calculated.

## Results

Xpert^®^ MTB/RIF assay: The assay detected 12 specimens as MTB with rifampicin- resistance. The resistance was conferred by four different *rpoB* gene mutations in the 81 bp-RRDR of MTB. These were detected by probes A, B, D, and E. The probe frequency associated with the observed rifampicin- resistance were as follows: E (7/12), B (3/12), A (1/12), D (1/12) and no rif-resistance was associated with probe C. No specimen yielded rifampicin resistance associated with more than one probe (mutation combinations). Probe D was associated with rifampicin monoresistance when compared with APM results, used as a reference standard. Middlebrook agar Proportion method: The APM results indicated that one (1/12), specimen was found to be rifampicin mono-resistant while the 11 were found to be MDR-TB. The former had been rifampicin resistant associated with probe D.

There was concordance of 100% for rifampicin resistance detection between Xpert^®^ MTB/RIF and Middlebrook 7H11 APM. Xpert^®^ MTB/RIF assay was found to have a concordance of 91.6% for MDR-TB case detection. The average contamination rates for MGIT and LJ throughout the three year period was 6.5 and 3%. Out of 313 Xpert positive specimens, three patients were not recovered by culture (MGIT and LJ combined). They were lost to contamination however two out of these three specimens had smear positive results from their processed concentrated specimens. We also report that culture had a sensitivity that was generally better than Xpert^®^ MTB/RIF assay. Concentrated smear microscopy was inferior to Xpert^®^ MTB/RIF assay [12].

## Discussion

The most common RRDR *rpoB* gene mutations in the 81 bp were in codons 531 (58%), 513 (25%), 526 (8%), 511 (8%), and none for codon 522. These were designated by probes E, B, D, A, and C respectively on the Xpert^®^ MTB/RIF assay. A study by Yue *et al*. found these frequencies 531 (41%), 526 (40%), and 513 (4%) in China [13]. Both studies conclude that codon 531 is the most prevalent associated with rifampicin resistance. In the previous studies, the sensitivity of the MTB/RIF test for detecting rifampicin resistance was 94.4 to 100% and the specificity was 98.3 to 100% [2, 14, 15]. When all the 12 rifampicin resistant isolates by Xpert^®^ MTB/RIF assay were subjected to the “gold standard” agar culture-based drug susceptibility testing (APM), therefore the assay had a concordance of 91.6% for MDR-TB detection. No rifampicin resistance discrepant results were observed between Xpert^®^ MTB/RIF and agar proportion method.

In our study, we did not identify any rifampicin resistance associated with probe C; probably this particular site of RRDR is less susceptible to mutations conferring this resistance, though we acknowledge that our sample size is small. However this may be explained by two possible reasons, it may mean that the selection pressure shaping probe C associated rifampicin resistance is absent in our setting and secondly the small sample size may have limited this likelihood. From this study, 4/12 MDR-TB (33.3%) patients said they had contacts previous with known TB patients though we could not ascertain the TB status of their index cases. This therefore puts a transmission rate of approximately 33% for these MDR-TB strains, more *in vivo* studies should be done to address the transmissibility of the MDR-TB strains.

In this study we found MDR-TB at 3.5%, WHO reported of increased MDR-TB detection in 2012 in the high TB burden countries. This was attributed to the application of Xpert^®^ MTB/RIF Assay [5]. Lukoye *et al*. reported MDR-TB prevalence to be 1.4% and further concluded that there was low relative prevalence of anti-TB drug resistance among new patients in Uganda than what WHO estimated [16]. Therefore the assay is a useful tool in the global fight against TB/MDR-TB. We had about 12% errors associated with Xpert^®^ MTB/RIF assay diagnostic software and Xpert^®^ MTB/RIF Assay G3 version 3 contributing more than half of the observed errors. With the upgrade to Xpert^®^ MTB/RIF assay G4 version 5, we recorded a remarkable reduction in errors/invalid results generated by the instrument.

## Conclusions

In conclusion, the Xpert^®^ MTB/RIF assay detected the rif-resistance associated mutations in RRDR 81 bp region. Out of the 12 rif-resistant isolates detected by this assay, 11 were MDR-TB by APM, and one was rifampicin mono-resistant and the profile of mutations in the 81-bp RRDR may be unique in these setting and probably can provide baseline data for rif-resistance patterns in the studied setting. Further studies should be done involving many MDR-TB strains isolated from these populations so that information about these mutations would be useful in development of novel therapies against TB disease. Much as the Xpert^®^ MTB/RIF assay is user-friendly and phenotypic anti-mycobacterial susceptibility testing methods remain an indispensable platform for drug susceptibility testing of MTB strains.

Further a combination of concentrated fluorescent smear microscopy and culture still remain useful in TB endemic settings since these provide an advantage of detecting MTB as well as isolating the nontuberculous mycobacteria which are gaining increasing medical importance causing both pulmonary and extrapulmonary lesions.

## Authors’ original submitted files for images

Below are the links to the authors’ original submitted files for images. Authors’ original file for figure 1 Authors’ original file for figure 2

## Abbreviations

MTB: *Mycobacterium tuberculosis*
RIF: rifampicin.
