# Supplementary figures and images for: Rifampicin resistance mutations in the 81 bp RRDR of rpoB gene in Mycobacterium tuberculosis clinical isolates using Xpert®MTB/RIF in Kampala, Uganda: a retrospective study
Source: BMC Infect Dis. 2014 Sep 4;14:481. doi: 10.1186/1471-2334-14-481 (PMC4164707; doi:10.1186/1471-2334-14-481)

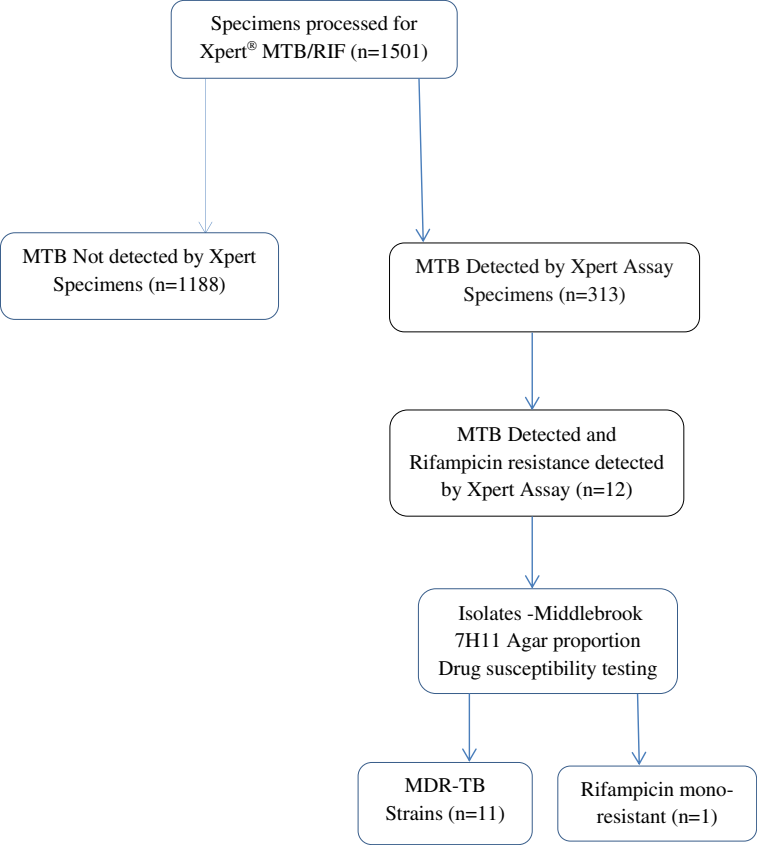

Supplement: Supplementary file 1 — Authors’ original file for figure 1 [file 12879_2014_3789_MOESM1_ESM.pdf]
