# Supplementary material for: Rifampicin resistance mutations in the 81 bp RRDR of rpoB gene in Mycobacterium tuberculosis clinical isolates using Xpert®MTB/RIF in Kampala, Uganda: a retrospective study
Source: BMC Infect Dis. 2014 Sep 4;14:481. doi: 10.1186/1471-2334-14-481 (PMC4164707; doi:10.1186/1471-2334-14-481)
Supplement: Supplementary file 2 — Authors’ original file for figure 2 [file 12879_2014_3789_MOESM2_ESM.docx]

Figure 2. For two Contact MDR-TB cases:

Case1. Probe E fails

Case 2. Probe D fails

From figure 2 above, given that these were two contact MDR-TB patients, these results show that these individuals have different MDR-TB strains as shown by the probes.
